# To specialize or too specialized?

**Published:** 2013-03-31

**Authors:** Daniel Mendelsohn

**Affiliations:** Schulich School of Medicine, University of Western Ontario, London, Ontario, Canada

In January of 2011 I, together with my fourth-year medical student colleagues, returned to the University of Western Ontario from our electives to begin four months of preparation for our medical licensing exam. During large group discussions on general clinical scenarios, it was clear that, for most students, specialization was well underway. When listing a differential diagnosis for a headache, the budding emergency physician saw a subarachnoid hemorrhage, the future family physician saw migraines, and the soon to be obstetrician/gynecologist saw preeclampsia. Before completing our “general practice” training, it was evident that many of us were already too committed to specialization to become general clinicians.

The 16 weeks of elective time we recently completed marked the first opportunity to customize our medical education. Students used their elective time to demonstrate a commitment to general practice or to their chosen specialties and to improve their knowledge of general medicine. Many students applying for Royal College specialty training programs had spent over half of their elective time (in some cases up to 12 weeks) on a single specialty service.

Since 1996, Canadian medical students apply through the Canadian Residency Matching Service in September of their 4^th^ year (or 3^rd^ year in a 3-year medical school curriculum)^1^. This tight timeline pressures many students applying to competitive specialties to decide on their specific career aspirations early on during their medical training, as applicants commonly perceive that residency program selection committees view a high proportion of electives in one specialty and specialty-specific research projects as appealing assets in a candidate. In many circumstances, students are committing to specialties prior to completing all their core clerkship rotations. The current system introduces two important conditions of great concern: 1) Students are required to commit to specialties too early, 2) Latter medical school training is now weighted towards specialization and away from mastering general medicine.

Until the 1990s, Canadian medical graduates entered a rotating internship before beginning general practice or applying for specialty programs.^2^ Advantages to the rotating internship included better familiarity with in-patient medicine and the ability to explore diverse fields of medicine prior to committing to a specialty.^3^ During the rotating internship era, 17.5% of Canada’s 1989 graduating class changed their choice of specialty after commencing residency.^4^ In Saskatchewan in 1994, 59% of survey respondents were practicing in a field different from the one they planned in their second-last year of medical school.^5^ However, in the 1980s, several reports recommended creating a different training program for family physicians instead of relying on the rotating internship. By 1993, all provinces had embraced a 2-year family medicine residency program and abandoned the rotating internship.^6^ Some Royal College programs then incorporated the rotating internship into their first year of training.^6^

Deciding on a specialty too early may lead to discontent with a resident’s chosen specialty. After abandoning the rotating internship, in 1995, 40% of graduating medical students felt poorly to moderately prepared to choose a residency and 7% of students reported they would like to change their chosen specialties after the match.^6^,^7^ The significant proportion of students who are unsure of their choice is especially concerning.

Given that the current residency application system is unlikely to change, medical schools should focus on providing students with a broad range of clinical exposures earlier in the first two years. In addition, clerkship programs should be structured to maximize students’ exposure to the major specialties prior to requiring commitment to a specific career.

Medical students use the final year of medical school to make themselves appealing candidates for residency programs. Specialty programs seem to prefer medical students who have already committed to specialization by dedicating a high proportion of their elective time to one field. The current system does not favor the applicant who is undecided (and perhaps open-minded) and wishes to use their elective time to explore various fields in medicine to facilitate a good decision. Abolishing the rotating internship created an environment where specialty candidates use the medical school experience as a means to gain entrance into specialty programs. The emphasis on becoming a competitive applicant for a specialty has diminished medical students’ opportunities to master general medicine. The current system raises a critical question: if residency programs train specialists, are medical schools already too specialized to focus on training general doctors first?
